# Spacer-Supported Thermal Ablation to Prevent Carbonisation and Improve Ablation Size: A Proof of Concept Study

**DOI:** 10.3390/biomedicines11020575

**Published:** 2023-02-16

**Authors:** Fiona Mankertz, Ole Gemeinhardt, Ute Felbor, Stefan Hadlich, Norbert Hosten

**Affiliations:** 1 Institute for Diagnostic Radiology and Neuroradiology, University Medicine Greifswald, 17475 Greifswald, Germany; 2 Department of Radiology, Charité—Universitätsmedizin Berlin, 10117 Berlin, Germany; 3 Institute for Human Genetics, University Medicine Greifswald, 17475 Greifswald, Germany

**Keywords:** thermal ablation, laser ablation, experimental radiology, ablation zone, Nd:YAG

## Abstract

Thermal ablation offers a minimally invasive alternative in the treatment of hepatic tumours. Several types of ablation are utilised with different methods and indications. However, to this day, ablation size remains limited due to the formation of a central non-conductive boundary layer. In thermal ablation, this boundary layer is formed by carbonisation. Our goal was to prevent or delay carbonisation, and subsequently increase ablation size. We used bovine liver to compare ablation diameter and volume, created by a stand-alone laser applicator, with those created when utilising a spacer between laser applicator and hepatic tissue. Two spacer variants were developed: one with a closed circulation of cooling fluid and one with an open circulation into hepatic tissue. We found that the presence of a spacer significantly increased ablation volume up to 75.3 cm^3^, an increase of a factor of 3.19 (closed spacer) and 3.02 (open spacer) when compared to the stand-alone applicator. Statistical significance between spacer variants was also present, with the closed spacer producing a significantly larger ablation volume (*p* < 0.001, M_Diff_ = 3.053, 95% CI[1.612, 4.493]) and diameter (*p* < 0.001, M_Diff_ = 4.467, 95% CI[2.648, 6.285]) than the open spacer. We conclude that the presence of a spacer has the potential to increase ablation size.

## 1. Introduction

The development of minimally invasive procedures has conferred the possibility of eliminating primary and secondary hepatic lesions. Over the last three decades, minimally invasive ablation has been established as a safe and effective alternative to conventional surgery. Tumour ablation is categorised in non-energy based ablation such as trans-arterial chemoembolisation (TACE) and energy-based ablation, the latter of which can be further separated into thermal and non-thermal ablation. Today, the most frequently used procedure is thermal ablation, with several well-documented and clinically studied methods such as radiofrequency ablation (RFA), microwave ablation (MWA) and laser ablation (LA). Whilst those methods differ in regard to their physical principles, they all share the same goal: the complete thermal ablation of tumorous tissue with an adequate margin of non-tumorous tissue. However, the maximum achievable ablation size has proven to be the greatest limitation for all those methods [1].

Ablation size (both in volume and diameter) is limited due to the transformation of viable energy-conducting tissue into a boundary layer which does not conduct energy efficiently. In thermal ablation, this process is known as carbonisation. Carbonisation occurs when tissue temperatures rise above 100 °C. As biological tissue is sensitive to external stress, a sudden increase in temperature leads to protein denaturation, the dissolution of hydrogen bonds and DNA polymerase inhibition [2]. At temperatures of over 45 °C, irreversible cell damage occurs. This process is sped up exponentially with further increases in temperature; it is the principle on which the thermal ablation of tumorous tissues relies on. However, when tissue temperatures exceed 100 °C, the affected tissue is charred and desiccated as gaseous microbubbles form and vaporisation occurs [3,4]. Carbonisation naturally develops primarily in zones with the highest tissue temperature. This translates to the central zone around the applicator in LA, around the needle electrodes in RFA [5] or around the microwave antennae [6].

The carbonised area around the ablation probe acts as an insulating border: a boundary layer. It hinders the effective diffusion of energy into peripheral tissue due to its physiological properties. This insulation consequently limits ablation size [4,7]. Additionally, in laser ablation photons are backscattered from the surrounding tissue onto the carbonised area. This further increases the temperature around the ablation probe [8]. As a result, damage to the laser fibre occurs.

Several innovations have been developed for delay or prevention, such as the introduction of an internal cooling system for both RFA and LA. This concept relies on a continuous diffusion of cooling fluid directly surrounding the probe, which lowers the central temperature and delays the formation of carbonisation for a limited time [9]. A more complicated but effective strategy is the reduction in arterial blood flow, and thus, the elimination of the heat sink effect. These strategies include total portal flow occlusion through the Pringle manoeuvre, angiographic balloon occlusion, as well as intraarterial embolisation [5]. The Pringle manoeuvre as well as other vascular clamping techniques have shown a greater efficacy in ablation size when additionally conducted. However, they harbour the risk of ischemia-reperfusion syndrome, especially in patients with hepatic steatosis or fibrosis of the liver [10,11]. The use of intermittent versus continuous clamping has been debated, both of which have their advantages and disadvantages regarding ischemia time and the risk of intraoperative haemorrhage [12].

An approach to preventing carbonisation is the use of a spacer. The implementation of a spacer in ablation therapy was expanded on in a different setting: the use of a spacer during focal prostate ablation has been proven to prevent heat damage to surrounding organs. In a 2021 study, Namakshenas et al. report that rectal temperatures were significantly lower when a polyethylene glycol spacer surrounding the laser applicator was utilised [13]. Yamaguchi et al. found in a 2023 study that the inclusion of a hydrogel spacer before hypofractioned irradiation reduced the number of fractions needed and increased the fraction size, resulting in more effective treatment [14]. Björeland et al. additionally noted that the implantation of a hyaluronic acid spacer in radiotherapy of prostate cancer significantly reduced genitourinary and gastrointestinal late-stage toxicity [15].

This spacer extends the distance between initial photon emission from the optical fibre and photon ingress into the targeted tissue. It additionally builds on the principle of the cooling system reducing central tissue temperatures to prevent carbonisation. We studied the impact of a spacer’s presence for laser ablation in an ex vivo liver model. Our aim was to facilitate comparison of ablation diameter and volume when using a spacer vs. a stand-alone applicator. As hepatic lesions between 25–50 mm are the most frequent [16] but current application methods rarely reach that threshold, we additionally investigated whether an ablation diameter of 50 mm could be attained. As the shapes created by ablation are elliptical as opposed to spherical, we reported longitudinal, perpendicular and vertical diameters.

## 2. Materials and Methods

### 2.1. Laser Ablation

We chose to use laser ablation to examine the impact of spacer-supported laser ablation on hepatic tissue coagulation size. Laser ablation, which is sometimes referred to as laser-induced thermal therapy (LITT), is optimal for this proof of principle study due to the clear visibility of carbonisation as a boundary layer. Carbonisation macroscopically appears as a distinct change in the colour of liver tissue. Additionally, coagulated non-viable tissue can easily and accurately be distinguished from viable tissue macroscopically. This was confirmed in a 2012 study by Hoffmann et al., in which was noted that in routine clinical thermal ablation, no histopathological sample is obtained to confirm the full ablation of tumorous tissue. Subsequently, they examined the relationship between peri-ablative invasive temperature measurements and post-ablative histopathological examination. They found that tissue confirmed as non-viable in 2,3,5-triphenyltetrazolium chloride viability staining could be macroscopically distinguished from viable tissue by its different colouring, as well as changes in tissue density. This macroscopically defined non-viable tissue also matched the necrotic areas found on H&E staining. In a 2011 study, Schneider et al. additionally found that non-viable pulmonal tissue following radiofrequency ablation could not be differentiated from viable tissue using standard H&E staining alone [17,18]. As such, we chose to forego histological staining due to the clear evidence of correlation with macroscopic findings.

**Figure 1:**
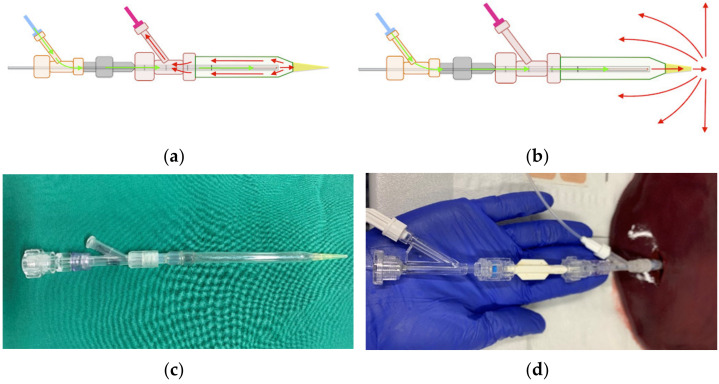
Diffusion circulation in the (**a**) closed spacer circuit and (**b**) open spacer circuit. Cooling fluid circulates through the spacer at a temperature of 20 °C and (**a**) drains into an external calorimeter or (**b**) diffuses into external tissue. In (**c**) is shown the real closed spacer prototype, as well as (**d**) the experimental setup using the closed spacer.

We conceptualised and produced a spacer in a miniseries for the purpose of conducting several experiments. The spacer augmented the distance between the laser’s optical fibre and tissue through the generation of a fluid-filled space. Two main variants of the spacer were created: one with a closed tip where diffusion fluid served as a cooling agent and circulated internally, as well as one with an open tip where the diffusion fluid was diffused into the surrounding hepatic tissue (see Figure 1).

Figure 1 shows the spacer with closed (a) and open (b) diffusion circulation. Isotone sodium chloride solution (0.9%) was used as a cooling agent. In a preceding experimental study, we found that there was no significant difference between the usage of sodium chloride and aqua destillata. Aqua destillata has the added disadvantage of its hypo-osmolar properties, which is less suitable for continuous infusion into the human body than 0.9% sodium chloride [15]. Additionally, Ishikawa et al. reported in 2013 that radiofrequency ablation produced significantly larger ablation zones when combined with the continuous infusion of saline solution. They also noted that when using a hypotone 50% glucose solution, ablation zones were diminished even in comparison to not infusing any fluid [14]. As such, we chose sodium chloride solution for its well-established compatibility with the human body and its efficacy in ablation therapy. Both spacers were filled with the cooling agent before tissue insertion, and fluid was diffused through means of a peristaltic pump at a set rate of 60 mL/h. The spacers were constructed from commercially available components: a glass pipette (B. Braun Melsungen, Melsungen, Germany) with a diameter of 4 mm, a distal 200 µL polypropylene pipette tip, as well as a proximal Y-connector (Figure 1c).

To ensure realistic ex vivo simulation, bovine livers (*n* = 15) were chosen due to their similarities to human liver in regard to capsule density and tissue structure. The livers were acquired from an abattoir (LandWert Hof, Stahlbrode, Sundhagen, Germany) <1 h post-slaughter and transported in a heat-resistant styrofoam container at room temperature. Pre-ablation, all livers were examined by a public health veterinarian. Any remains of adjunct structures and omental fat were removed to ensure tissue homogeneity. Large vessels such as portal and caval veins, hepatic arteries, or the biliary duct were longitudinally incised to prevent fluid diffusion into air-filled vessels. Any hepatic tissue measured to be <100 mm in diameter was also removed. The stand-alone applicator or spacer-supported applicator were first filled with commercial saline solution and then inserted into hepatic tissue up to the proximal Y-connector (Figure 1d).

The process of laser ablation with a commercially available applicator system (RoweMed, Parchim, Germany) has been documented in several studies and was utilised according to standard practice [9]. The term “stand-alone applicator system” refers to the conglomerate of optical fibre enveloped by a diffusion catheter and a proximal Y-connector. For the purpose of our study, we used a Medilas Fibertom 5100 laser with a Nd:YAG 1064 nm wavelength (Dornier Medtech Europe GmbH, Munich, Germany).

In order to determine the maximal wattage we could use without the occurrence of carbonisation, the laser’s power was increased from 15 W to 35 W in 5 W increments at a set time interval of 10 min until carbonisation was recorded. Subsequently, the lasing time interval was increased from 5 min to 30 min in 5 min increments at a set wattage of 25 W until carbonisation was recorded.

For the stand-alone applicator system, the maximum combination of wattage and time interval was 25 W and 10 min. For the spacer-supported applicator systems, the maximum combination was 25 W and 25 min. To facilitate comparison between not only the stand-alone applicator system and the spacers, but also between the spacers, we conducted an *interspacial* trial with all three experimental designs at 25 W and 10 min, as well as an *intraspacial* trial with both spacer variants at 25 W and 25 min.

**Figure 2:**
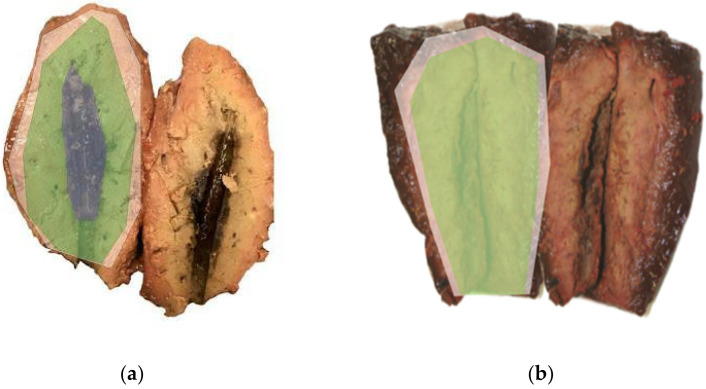
Cross section of the ablation zones in (**a**) the stand-alone applicator (25 W; 10 min) and (**b**) the spacer-supported applicator (25 W; 25 min). Black overlay: carbonisation zone. Green overlay: non-viable tissue. White overlay: total ablation zone (“red zone”).

At the end of each LA procedure, the tissue was sliced along the antenna tract, revealing a cross section of the ablation zone. Ablation zones are defined by their cell viability, as correlated by vitality staining and standard H&E staining in external studies [17,18]. The central zone (black overlay in Figure 2a) is the carbonised boundary layer of charred, non-conducive tissue. The proximal zone of ablation (green overlay in Figure 2a,b) is comprised of fully ablated, non-viable tissue. The distal zone (white overlay in Figure 2a,b) has both viable and non-viable tissue. Gemeinhardt et al. also separated the zone of partial ablation into a “red zone 1”, in which vital and non-vital cells comprise a similar percentage, as well as a “red zone 2” in which cells are mostly vital [19]. All three ablation zones were measured, and their longitudinal, perpendicular and vertical diameter was recorded.

### 2.2. Volumetry

As MRI imaging is the standard method for periprocedural thermometry and postprocedural monitoring for laser ablation, post-ablation scans were obtained on a 7T. ClinScan 70/30 Biospec MRI USR (Bruker BioSpin GmbH, Bruker Scientific Instruments, Ettlingen, Germany). T1 weighted FL2D imaging was performed due to its superior differentiation of signal loss caused by tissue coagulation. Coagulation presented as hyperintense in comparison to liver tissue. Volumetry was conducted through a standard DICOM viewer. Sagittal and horizontal sequences were analysed, and closed-polygon area was calculated. Following a conclusive area selection, tissue coagulation volume was calculated through ROI volumetry (V_MRI_).

Additionally, tissue coagulation volume was manually measured through displacement volumetry (V_D_). Displacement volumetry relies on Archimedes’ principle that the volume of a buoyant submerged object is equal to the weight of the fluid displaced by the object (m = V). Post-MRI, the ablated tissue was debrided into an ellipsoid shape, and the mass of the water displaced by the ellipsoid tissue was weighed (1 g = 1 cm^3^). Post-MRI DICOM volumetry was compared with displacement volumetry to prevent type I errors.

As seen in Table 1, we found that there was no significant difference between the two methods of volumetric analysis, as the level of statistical significance was consistently >0.05. We thus chose to proceed to measure tissue coagulation volume through displacement volumetry. MRI volumetry may be utilised in later studies in the knowledge that it accurately represents non-viable tissue boundaries.

### 2.3. Statistics

The statistical analysis was performed through IBM SPSS (v28.0.1; Chicago, IL, USA). To ascertain the reliability of the obtained results, the Intraclass Correlation Coefficient (ICC) was calculated. Both data sets from the interspacial and intraspacial trials were then analysed through a one-way repeated measures multivariate analysis of variance (orMANOVA). The orMANOVA analysis included the determination of multivariate normality through Shapiro–Wilks testing, the presence of statistical significance using Wilks’ Lambda testing and, finally, the quality of present statistical significance (*p* > 0.05) through post hoc Games-Howell testing.

## 3. Results

When comparing all three groups (stand-alone applicator S_N_, closed spacer circuit S_C_ and open spacer circuit S_O_) at a time interval of 10 min, S_N_ achieved a significantly higher perpendicular ablation diameter when compared to S_C_ (*p* < 0.001, (M_Diff_ = 8.9333, 95% CI[7.6280, 10.2387]) and S_O_ (*p* < 0.001, (M_Diff_ = 6.6000, 95% CI[5.2947, 7.9053]). S_N_ also achieved a significantly higher ablation volume when compared to Sc (*p* < 0.001, (M_Diff_ = 5.4887, 95% CI[4.4291, 6.5482]) and S_O_ (*p* < 0.001, (M_Diff_ = 5.1267, 95% CI[4.0671, 6.1682]). However, for S_N_, any increase in the ablation time interval above 10 min resulted in carbonisation, damage to the laser’s optical fibre and activation of the light protection system. As this was only the case for S_N_ and an increase in ablation time interval was possible without complications for S_C_ and S_O_, we decided to compare the two spacer variants at a time interval of t = 25 min. Both spacers were able to complete a time interval of 25 min without any damage to the optical fibre or carbonisation.

At a time interval of t = 25 min, both S_C_ and S_O_ achieved significantly higher ablation diameter and volume than S_N_. S_C_’s perpendicular diameter Ø_P_ was >50 mm (see Table 2). Likewise, S_C_ achieved a significantly higher perpendicular ablation diameter (*p* < 0.001, (M_Diff_ = 4.46667, 95% CI[2.6479, 6.2854]) and ablation volume (*p* < 0.001, (M_Diff_ = 3.0527, 95% CI[1.6121, 4.4932]) than S_O_.

We calculated an increase in ablation volume by the factor of 3.18 for the closed spacer-supported applicator and by the factor of 3.06 for the open spacer-supported applicator.

ICC estimates and their 95% confidence intervals were calculated based on a mean-rating (k = 25), absolute-agreement, two-way mixed-effects model. The data set recording V_MRI_ and V_D_ resulted in an average ICC of 0.85. This indicates good reliability. Based on the 95% confidence interval of the average, moderate-to-excellent reliability and internal consistency can be assumed. Cronbach’s alpha, another measure of reliability, resulted as 0.85. This indicates good internal consistency. Multivariate normality cannot be directly tested through most means; therefore, univariate normality was tested under the assumption that it would serve as an estimate of the presence of multivariate normality [20]. All groups were normally distributed across both dependent variables, as assessed by the Shapiro–Wilk test (α = 0.05). The presence of statistical significance was calculated through Wilks’ Lambda to be *p* < 0.001 for all dependent variables. Following this, post hoc univariate ANOVAs were conducted and determined a statistically significant difference for all dependent variables as the effect of the spacer category:

Ø_P_: F(2,42) = 148.735, ***p* < 0.001**, partial η^2^ = 0.876

V_MRI_: F(2,42) = 82.057, ***p* < 0.001**, partial η^2^ = 0.796

V_D_: F(2,42) = 99.085, ***p* < 0.001**, partial η^2^ = 0.825

Finally, a Games-Howell post hoc analysis was performed to ascertain the quality of statistical significance. Pairwise differences in ablation volume and perpendicular diameter were also found to be statistically significant.

## 4. Discussion

Over the past two decades, thermal ablation and its different energy sources have been established as a valid treatment option of hepatic lesions, specifically hepatocellular carcinoma and metastases. While different types of thermal ablation such as RFA, MWA and LA differ by practicability, cost efficiency, as well as absolute and relative contraindications, a main limitation of each method remains its achievable tissue coagulation size. Small lesions up to 30 mm can be fully ablated when including a safe margin [21], while medium-sized lesions with a diameter of 30–50 mm are unable to be fully ablated with a single applicator in only one application [1]. The full ablation of tumours between 30–50 mm is only possible through multiple applicators or several rounds of applications and differing applicator positioning, as well as the addition of surgical and interventional techniques such as vascular clamping [2] and TACE [1]. Vascular clamping of the total, partial or selective hepatic flow is a valid method. However, it is technically demanding, shows association with intestinal congestion and requires an intermittent or continuous period of organ ischemia [10,11].

The size limitation in thermal ablation is caused by overheating near the thermal applicator, which at a certain point transforms viable heat-conducting tissue into a non-conductive boundary layer. This boundary layer acts as an insulating “shell”, which limits further energy transmission into peripheral tissue. In RFA, abrupt changes in tissue impedance have been reported to create “heat spots” around the probes [22]. In MWA and LA, the formation of a boundary layer has been described as carbonisation around the applicators [23]. Preventing carbonisation has been key in increasing ablation size, as several developments in recent years show: For MWA in recent years, an internal cooling jacket has been developed [24]. RFA and LA have been improved through the standardised development of cooling diffusers [25,26]. These improvements share similar aims and methods; their goal is the increase in energy diffusion through the targeted tissue by decreasing the temperature immediately adjacent to the heat source.

A second approach to maximise ablation size is the usage of higher wattage and longer ablation times. As the output and transmission of energy is directly linear to power input and time interval E=P·t, conducting an ablation protocol with higher power would result in higher ablation volume and diameter. However, when power is set too high, central tissue temperatures rise exponentially and carbonisation occurs much faster [27]. In our preliminary studies, to determine optimal wattage, we noted that any power above 25 W would lead to almost instantaneous abortion of the ablation process. Conversely, low power, such as 5 W or 10 W, would allow longer ablation time intervals, but ultimately resulted in a smaller ablation size. We thus selected a power input of 25 W.

A technically comparatively simple and cost-effective approach to prevent these thermal changes of tissue is the usage of a spacer. It creates an artificial, fluid-filled space between the applicator and the targeted, viable tissue. The concept behind the spacer’s efficacy has not been fully explained up to this point; however, there are two hypotheses.

- The hypothesis of **optical gain**: photons travel through the spacer’s fluid medium before entering hepatic tissue. As the absorption coefficient in the liquid medium is low, the laser’s stimulated emission is not absorbed until it enters hepatic tissue. In hepatic tissue, the absorption coefficient rises sharply, and photothermal interactions take place after absorption [28].
- The hypothesis of **heat diffusion**: similar to how the physiological heat sink effect limits ablation size close to larger vessels [29], the constant diffusion of fluid through the spacer prevents the tissue closest to the spacer borders from carbonising. This translates to further photon diffusion, and thus, broader thermal ablation and tissue coagulation size.

In our study, we compared whether a cooled spacer with or without fluid diffusion into the targeted tissue enlarged ablation volume when compared to a stand-alone applicator. Our aim was to observe the impact on the spacer’s presence on tissue coagulation diameter and volume. This was achieved through laser ablation. The advantage of laser ablation is the clear highlighting of carbonisation, fully non-viable “white zone” tissue and partially viable “red zone” tissue [19].

A statistically significant increase in tissue coagulation volume and diameter was achieved through both spacer variants compared to the stand-alone applicator. For the stand-alone applicator, a maximum volume of 23.6 cm^3^ could be ablated. These results simulate experiments conducted by Vogl et al., who managed a maximum ablation volume of 22.4 cm^3^ with an internally cooled power laser [26]. Similar to previous studies by Hosten et al., the stand-alone applicator was able to complete the ablation process if the time interval was set at 10 min (see Figure 2a). At longer time intervals, we recorded tissue carbonisation and damage to the optical fibre. At the same time, we recorded that spacer-supported ablation processes could be prolonged to up to 25 min without tissue carbonisation (see Figure 2b). The attained tissue coagulation volume for the spacer with a closed circulation (S_C_) allowed the mean ablation of 75 cm^3^, as well as a white zone diameter of fully non-viable tissue of >50 mm in the smaller axis perpendicular to the applicator. When compared to the stand-alone applicator, we thus recorded an improvement in ablation volume of 319% for the closed spacer prototype and of 302% for the open spacer prototype.

Not only was the mean ablation diameter and volume a significant increase from results obtained by the stand-alone applicator, the results were also significant when evaluating the spread: S_C_ reached the threshold of 50 mm ablation diameter in 12/15 cases (80%). In 3/15 cases it exceeded the threshold by ≥5 mm (20%), which is recognised as the minimal tumour margin considered “safe” in ablation procedures [30]. S_O_ reached the threshold of 50 mm ablation diameter in 4/15 cases (26.67%), but failed to exceed the threshold of ≥5 mm. However, the open spacer also opens several possibilities. The spread of fluid into nearby tissue resembles the method used in trans-arterial chemoembolization (TACE). None of the studies reporting a TACE-LITT combination therapy of hepatic tumour tissue mention the possibility of a simultaneous application of both thermal ablation and chemical ablation [31,32]. However, when considering the options of the open-ended spacer, a replacement of the sodium chloride used in this trial by cytotoxic substances such as alcohol (PAI) or doxorubicin (TACE) seems feasible.

Our thermal ablation was performed through laser ablation. As radiofrequency ablation and microwave ablation have been the objects of extensive research and comparative studies, the relevancy of using a laser-based thermal ablation must be considered. However, the main limitation of developing a boundary layer of non-conductive tissue is a shared principle among RFA, MWA and LA. Hui et al. name the pathophysiological process of “tissue charring” as a limitation in RFA. This process results in raised tissue impedance and decreased temperatures in peripheral tissue. They substantiate this charring with the ablation of both surrounding larger vessels, as well as the destruction of capillary microperfusion [33]. Various papers also apply the term of carbonisation to RFA [19,34,35]. For MWA, Jin et al. found that tissue around the applicator antennae during MWA is similarly affected. The direct deposit of energy into central tissue causes a severe centrifugal temperature gradient, resulting in the desiccation and carbonisation of tissue. The authors name anti-carbonisation strategies such as the development of a cooling jacket as one of the top priorities of MWA research [6].

As the generation of a boundary layer of non-conductive tissue remains the underlying mechanism behind carbonisation, desiccation and “tissue charring”, laser ablation is not inherently ill-suited for this study. It is technically simple to use in experiments, does not require the application of grounding pads, and its results can be easily monitored through post-interventional MRI [36]. Therefore, it is not only applicable, but also qualified for a proof of principle study such as this one. Laser ablation can be considered optimal for preclinical studies, yet it also performs worse in terms of ablation size when compared to radiofrequency ablation and microwave ablation. Radiofrequency ablation is deemed a standard staple in minimally invasive tumour therapy, yet it also has several limitations: multiple probes cannot be used due to electromagnetic interference, its heating patterns are more heterogenous and unpredictable compared to microwave ablation, and the presence of grounding pads increases the risk of periprocedural burns [37]. Higher intratumoral temperatures in microwave ablation are considered its main disadvantage. Subsequently, the further development of a method to circumvent these temperature spikes has been studied in recent years [38]. Observing the influence of a spacer’s presence in microwave ablation and its impact on tissue temperature and ablation size is a natural next step.

This study is a prototypical proof of concept experiment. As such, it differs from a clinical study in three main aspects: the setting was (a) ex vivo, (b) conducted with bovine liver and (c) healthy hepatic tissue. Ex vivo studies cannot fully replicate the effects of thermal ablation in perfused human tissue and are thus limited in their clinical significance. Influencing factors include the heat sink effect of nearby vascular structures and ablation zone expansion through the perivascular extension of heat damage [38,39]. Before clinical experiments using target patients with liver lesions or neoplasms, it is indubitable that more experiments testing the effect of spacer-supported thermal ablation in bovine as well as human tissue, should be conducted. This study thus serves as a first step. Further, the spacer developed was manufactured with commercially available components such as a glass pipette and a polypropylene pipette tip. The spacer body’s pipette diameter was arbitrarily chosen. To what extent the spacer’s diameter influences the tissue coagulation volume and diameter was not examined and cannot be derived from this study.

In an extension of the experiment, we tested whether tissue dissection and conversion of the water weight displaced by the ablated tissue into the tissue’s volume itself (1 g = 1 cm^3^) was equivalent to post-ablation MR-imaging. We found no statistical significant differences (*p* = 0.9400, 95% CI −1.7832 to 1.9206) when comparing V_MRI_ and V_D_. As tissue dissection and manual weighing is not possible in vivo, we therefore conclude that MR-imaging is a viable alternative.

We conclude that the presence of a spacer significantly improves ablation volume and diameter, as well as prevents the formation of a boundary layer. This proof of concept study could be followed up in the future and applied to other methods of thermal ablation, such as radiofrequency ablation and microwave ablation. A spacer constructed for usage in microwave ablation would necessarily be composed of heat-resistant, highly conductive materials with low absorption. Before the utilisation of the spacer in clinical practice is realised, further evaluation of its efficacy and safety is needed. This may be accomplished by means of continued preclinical studies on in vivo models. Spacer-supported thermal ablation is a viable option to be developed further.

## Figures and Tables

**Table 1:** Calculated *p*-value between V_MRI_ and V_D_.

Comparisons	Two-Tailed *p* Value between V_MRI_ and V_D_	Mean Difference	95% Confidence Interval of Difference
S_N_ (25 W 10 min)	0.9228	−0.0507	−1.1126 to 1.0112
S_C_ (25 W 10 min)	0.1216	0.5507	−0.1558 to 1.2572
S_O_ (25 W 10 min)	0.1302	0.6327	−0.1985 to 1.4638
S_C_ (25 W 25 min)	0.9400	0.0687	−1.7832 to 1.9206
S_O_ (25 W 25 min)	0.8328	−0.1040	−1.1035 to 0.8955

V_MRI_: ROI volumetry; V_D_: displacement volumetry; S_N_: stand-alone applicator; S_C_: closed spacer-supported applicator; S_O_: open spacer-supported applicator.

**Table 2:** Mean ablation diameter and volume are increased by use of a spacer (S_C_ und S_O_) in comparison to the stand-alone applicator (S_N_). *n* = 15.

	Power [W]	Time [min]	Ø_L_ [mm]	Ø_P_ [mm]	Ø_V_ [mm]	V_D_ [cm^3^]
Stand-alone applicator (S_N_)	25	10	49.533	37.600	12.650	23.613
Closed spacer-supported applicator (S_C_)	25	25	65.800	52.067	21.950	75.253
Open spacer-supported applicator (S_O_)	25	25	58.333	47.600	26.000	72.201

Ø_L_ = Longitudinal diameter measured along the fibre axis. Ø_P_ = Perpendicular diameter measured across the fibre axis. Ø_V_ = Vertical diameter measured across the fibre axis. V_D_ = Displacement volumetry.

## Data Availability

A full data set is available upon request to the corresponding author.

## References

[1] Lucatelli P. Ablation: Is There Still Room for Improvement [Lecture Recording]. ECIO 2022. https://library.cirse.org/ecio2022/crs/101-3-ablation-is-there-still-room-for-improvement.

[2] Poch F.G.M., Neizert C.A., Geyer B., Gemeinhardt O., Niehues S.M., Vahldiek J.L., Bressem K.K., Lehmann K.S. (2021). Perivascular vital cells in the ablation center after multibipolar radiofrequency ablation in an in vivo porcine model. Sci. Rep..

[3] Sturesson C. (1998). Interstitial laser-induced thermotherapy: Influence of carbonization on lesion size. Lasers Surg. Med..

[4] Hong K., Georgiades C. (2010). Radiofrequency ablation: Mechanism of action and devices. J. Vasc. Interv. Radiol..

[5] Rhim H., Goldberg S.N., Dodd G.D., Solbiati L., Lim H.K., Tonolini M., Cho O.K. (2001). Essential techniques for successful radio-frequency thermal ablation of malignant hepatic tumors. Radiographics.

[6] Jin X., Feng Y., Zhu R., Qian L., Yang Y., Yu Q., Zou Z., Li W., Liu Y., Qian Z. (2022). Temperature control and intermittent time-set protocol optimization for minimising tissue carbonization in microwave ablation. Int. J. Hyperth..

[7] Singh S., Repaka R. (2017). Temperature-controlled radiofrequency ablation of different tissues using two-compartment models. Int. J. Hyperth..

[8] Jacques S. (1998). Continuous Laser Ablation of Carbonized Tissue: Simple Rules. NewsEtc. https://omlc.org/news/may98/ablation/index.html.

[9] Hosten N., Stier A., Weigel C., Kirsch M., Puls R., Nerger U., Jahn D., Stroszczynski C., Heidecke C.D., Speck U. (2003). Laser-induzierte Thermotherapie (LITT) von Lungenmetastasen: Beschreibung eines miniaturisierten Applikators, Optimierung und erste Patientenbehandlungen [Laser-induced thermotherapy (LITT) of lung metastases: Description of a miniaturized applicator, optimization, and initial treatment of patients]. RöFo.

[10] Ercolani G., Ravaioli M., Grazi G.L., Cescon M., Del Gaudio M., Vetrone G., Zanello M., Pinna A.D. (2008). Use of vascular clamping in hepatic surgery: Lessons learned from 1260 liver resections. Arch. Surg..

[11] Chouillard E.K., A Gumbs A., Cherqui D. (2010). Vascular clamping in liver surgery: Physiology, indications and techniques. Ann. Surg. Innov. Res..

[12] Lesurtel M., Lehmann K., De Rougemont O., Clavien P.A. (2009). Clamping techniques and protecting strategies in liver surgery. HPB.

[13] Namakshenas P., Mojra A. (2021). Optimization of polyethylene glycol-based hydrogel rectal spacer for focal laser ablation of prostate peripheral zone tumor. Phys. Med..

[14] Ishikawa T., Kubota T., Horigome R., Kimura N., Honda H., Iwanaga A., Seki K., Honma T., Yoshida T. (2013). Radiofrequency ablation during continuous saline infusion can extend ablation margins. World J. Gastroenterol..

[15] Pestana C. (2000). Fluids and Electrolytes in the Surgical Patient.

[16] Wu G., Wu J., Wang B., Zhu X., Shi X., Ding Y. (2018). Importance of tumour size at diagnosis as a prognostic factor for hepatocellular carcinoma survival: A population-based study. Cancer Manag. Res..

[17] Hoffmann C.O.M., Rosenberg C., Linder A., Hosten N. (2012). Residual tumor after laser ablation of human non-small-cell lung cancer demonstrated by ex vivo staining: Correlation with invasive temperature measurements. MAGMA.

[18] Schneider T., Reuss D., Warth A., Schnabel P.A., Von Deimling A., Herth F.J., Dienemann H., Hoffmann H. (2011). The efficacy of bipolar and multipolar radiofrequency ablation of lung neoplasms-results of an ablate and resect study. Eur. J. Cardiothorac. Surg..

[19] Gemeinhardt O., Poch F.G., Hiebl B., Kunz-Zurbuchen U., Corte G.M., Thieme S.F., Vahldiek J.L., Niehues S.M., Kreis M.E., Klopfleisch R. (2016). Comparison of bipolar radiofrequency ablation zones in an in vivo porcine model: Correlation of histology and gross pathological findings. Clin. Hemorheol. Microcirc..

[20] Hemmerich W. (2015). Entscheidungshilfe für Statistische Verfahren. Wiesbaden, Germany. https://statistikguru.de/test-entscheidungshilfe/dtCp.html.

[21] Sartori S., Di Vece F., Ermili F., Tombesi P. (2017). Laser ablation of liver tumors: An ancillary technique, or an alternative to radiofrequency and microwave?. World J. Radiol..

[22] Payne M., Bossmann S.H., Basel M.T. (2020). Direct treatment versus indirect: Thermo-ablative and mild hyperthermia effects. Wiley Interdiscip. Rev. Nanomed. Nanobiotechnol..

[23] Amabile C., Farina L., Lopresto V., Pinto R., Cassarino S., Tosoratti N., Goldberg S.N., Cavagnaro M. (2017). Tissue shrinkage in microwave ablation of liver: An ex vivo predictive model. Int. J. Hyperth..

[24] Wang Y., Sun Y., Feng L., Gao Y., Ni X., Liang P. (2008). Internally cooled antenna for microwave ablation: Results in ex vivo and in vivo porcine livers. Eur. J. Radiol..

[25] Shi X., Pan H., Ge H., Li L., Xu Y., Wang C., Xie H., Liu X., Zhou W., Wang S. (2019). Subsequent cooling-circulation after radiofrequency and microwave ablation avoids secondary indirect damage induced by residual thermal energy. Diagn. Interv. Radiol..

[26] Vogl T.J., Mack M.G., Roggan A., Straub R., Eichler K.C., Muller P.K., Knappe V., Félix R. (1998). Internally cooled power laser for MR-guided interstitial laser-induced thermotherapy of liver lesions: Initial clinical results. Radiology.

[27] Gough-Palmer A.L., Gedroyc W.M. (2008). Laser ablation of hepatocellular carcinoma—A review. World J. Gastroenterol..

[28] Germer C.T., Roggan A., Ritz J.P., Isbert C., Albrecht D., Müller G., Buhr H.J. (1998). Optical properties of native and coagulated human liver tissue and liver metastases in the near infrared range. Lasers Surg. Med..

[29] Kim C. (2018). Understanding the nuances of microwave ablation for more accurate post-treatment assessment. Future Oncol..

[30] Laimer G., Jaschke N., Schullian P., Putzer D., Eberle G., Solbiati M., Solbiati L., Goldberg S.N., Bale R. (2021). Volumetric assessment of the periablational safety margin after thermal ablation of colorectal liver metastases. Eur. Radiol..

[31] Pacella C.M., Bizzarri G., Magnolfi F., Cecconi P., Caspani B., Anelli V., Bianchini A., Valle D., Pacella S., Manenti G. (2001). Laser thermal ablation in the treatment of small hepatocellular carcinoma: Results in 74 patients. Radiology.

[32] Pacella C.M., Bizzarri G., Cecconi P., Caspani B., Magnolfi F., Bianchini A., Anelli V., Pacella S., Rossi Z. (2001). Hepatocellular carcinoma: Long-term results of combined treatment with laser thermal ablation and transcatheter arterial chemoembolization. Radiology.

[33] Hui T.C., Kwan J., Pua U. (2021). Advanced Techniques in the Percutaneous Ablation of Liver Tumours. Diagnostics.

[34] Brieger J., Pereira P.L., Trübenbach J., Schenk M., Kröber S.-M., Schmidt D., Aubé C., Claussen C.D., Schick F. (2003). In vivo efficiency of four commercial monopolar radiofrequency ablation systems: A comparative experimental study in pig liver. Investig. Radiol.

[35] Poch F.G.M., Neizert C.A., Gemeinhardt O., Geyer B., Eminger K., Rieder C., Niehues S.M., Vahldiek J., Thieme S.F., Lehmann K.S. (2018). Intermittent Pringle maneuver may be beneficial for radiofrequency ablations in situations with tumor-vessel proximity. Innov. Surg. Sci..

[36] Vogl T.J., Naguib N.N.N., Eichler K., Lehnert T., Ackermann H., Mack M.G. (2008). Volumetric evaluation of liver metastases after thermal ablation: Long-term results following MR-guided laser-induced thermotherapy. Radiology.

[37] Poulou L.S., Botsa E., Thanou I., Ziakas P.D., Thanos L. (2015). Percutaneous microwave ablation vs radiofrequency ablation in the treatment of hepatocellular carcinoma. World J. Hepatol..

[38] Ozen M., Raissi D. (2022). Current perspectives on microwave ablation of liver lesions in difficult locations. J. Clin. Imaging Sci..

[39] Singh S., Siriwardana P.N., Johnston E.W., Watkins J., Bandula S., Illing R., Davidson B.R. (2018). Perivascular extension of microwave ablation zone: Demonstrated using an ex vivo porcine perfusion liver model. Int. J. Hyperth..

